# Consumption of Cashew Nuts on Oxidative Stress and Neuroinflammation Markers in Methionine‐Induced Hyperhomocysteinemia in Rats

**DOI:** 10.1002/fsn3.71714

**Published:** 2026-04-09

**Authors:** Rosalba Siracusa, Alessia Arangia, Regina Aparo, Marika Cordaro, Roberta Fusco, Giuseppina Mandalari, Salvatore Cuzzocrea, Daniela Impellizzeri, Rosanna Di Paola, Ramona D'Amico

**Affiliations:** ^1^ Department of Chemical, Biological, Pharmaceutical and Environmental Sciences University of Messina Messina Italy; ^2^ Department of Biomedical Dental and Morphological and Functional Imaging University of Messina Messina Italy; ^3^ Link Campus University of Roma Roma Italy; ^4^ Department of Veterinary Sciences University of Messina Messina Italy; ^5^ Department of Medicine and Surgery “Kore” University of Enna Enna Italy

**Keywords:** cashew nuts, hyperhomocysteinemia, neuroinflammation, oxidative stress

## Abstract

Hyperhomocysteinemia (HHcy), a metabolic disorder that causes a higher risk of neurovascular disease, is brought on by elevated blood levels of Hcy. Increased production of ROS and neuroinflammation, which result in neuronal damage and ultimately neuronal death, are known consequences of HHcy. The oxidative effect could be neutralized through the consumption of products rich in polyphenols, such as cashew nuts. Therefore, the objective of the study was to investigate the beneficial effect of cashews in an experimental condition of HHcy. HHcy was induced in rats by oral methionine (Meth) administration for 30 days, and cashew nuts at the dose of 100 mg/kg were administered by oral gavage for 30 consecutive days. Our results showed that Meth administration induced oxidative stress, astrocytes, and microglia activation, and neuronal cell death. Daily consumption of cashew nuts was able to counteract oxidative stress by the modulation of the antioxidant NRF‐2 pathway and consequent reduction of lipid peroxidation and upregulation of antioxidant enzyme levels, such as GSH and HO‐1. At the same way, Cashew nuts reduced neuroinflammatory markers and apoptotic process, as demonstrated by TUNEL assay and Bax and Bcl‐2 levels. Thus, the results suggested that the balanced consumption of cashew nuts could have a positive action in the prevention of HHcy‐induced disorders.

## Graphical abstract

Methionine‐induced hyperhomocysteinemia promotes oxidative stress, neuroinflammation, and neuronal damage in the brain. Oral administration of cashew nuts activates the Nrf2 pathway, reducing lipid peroxidation, glial activation, and apoptosis, ultimately leading to neuroprotection.

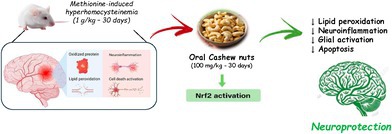

## 1 Introduction

Homocysteine (Hcy) is an intermediate amino acid formed during the metabolism of methionine. Since food contains little to no Hcy, methionine found in plant and animal proteins provides nearly all of the body's Hcy (Çelik et al. 2017). There are two main ways that Hcy can be metabolized: the transsulfuration pathway converts roughly 50% of it to cysteine, while the other 50% undergoes remethylation to generate methionine (Selhub and Miller 1992). High blood levels of Hcy are known as hyperhomocysteinemia (HHcy), and they are associated with a higher risk of neurovascular disease (Fu et al. 2018). In fact, HHcy appears to increase the permeability of the blood–brain barrier by causing damage to the cell junction of brain microvascular endothelial cells (Fu et al. 2018; Qureshi et al. 2005). More specifically, the excitatory amino acid Hcy increases the susceptibility of neuronal cells to oxidative and excitotoxic damage (Kamat et al. 2013). The literature indicates that the risk of neurological disorders is positively and dose‐dependently correlated with a mild to moderate increase in plasma Hcy levels (Herrmann and Obeid 2011). Therefore, even if the exact systems guiding these events are still unknown, HHcy plays a crucial part in the pathophysiology of many neurological system disorders, such as Parkinson's disease, stroke, epilepsy, dementia, multiple sclerosis, and Alzheimer's disease. It is commonly known that high levels of Hcy lead to increased generation of reactive oxygen species (ROS), which not only have detrimental effects on the cardiovascular system but also have an adverse effect on the brain, causing neuronal injury and ultimately resulting in the death of neurons (Ientile et al. 2010). Other mechanisms that associate the effects of HHcy with cellular damage in the neurological are connected to inflammatory processes. Indeed, Hcy can activate microglia and astrocytes which in turn trigger an inflammatory response that causes neuronal death (Chen et al. 2017). Additionally, mildly elevated levels of Hcy may increase the risk of vascular dementia, Parkinson's disease‐related dementia, and multiple sclerosis‐related cognitive decline (Reynolds 2017, 2006). Furthermore, clinical research has shown a linkage between HHcy and cognitive impairment in Alzheimer's disease, as well as a link between high plasma Hcy levels and the likelihood of cognitive decline (Nie et al. 2014). It has been shown that the consumption of folic acid, vitamins B2, B6, and B12 slowed the cognitive impairment (Smith et al. 2010). Therefore, diet and the consumption of natural substances, thanks to the presence of bioactive compounds, can help to modulate the response to inflammation and oxidative stress, and in this way counteract the progression of HHcy. Flavonoids are a class of naturally occurring substances with potential medicinal qualities that are typically found in plants as secondary metabolites (Çelik et al. 2017). Plants, fruits, and nuts that are high in phenolic compounds are particularly well known for their many biological properties, such as anti‐inflammatory, anti‐allergic, antiviral, antibacterial, and antitumoral effects (Impellizzeri et al. 2022; Sun and Shahrajabian 2023; Rudrapal et al. 2022; Fusco, Siracusa, et al. 2020). In comparison to a typical healthy diet, a diet high in walnuts lowers serum cholesterol levels, according to a number of research on nuts (Dias et al. 2019). Specifically, when included in a well‐balanced diet, cashew nuts can lower the risk of metabolic syndrome and cardiovascular disease, especially stroke (Liu et al. 2018; Bonifácio et al. 2023). These protective effects are probably due to the high content of flavonoids, anthocyanins, folates, and unsaturated fatty acids present in cashews. In fact, the antioxidant properties of these compounds are directed toward hydroxyl and superoxide radicals, therefore capable of counteracting the oxidative degradation of ROS (Cordaro, Fusco, et al. 2020; Fusco, Cordaro, et al. 2020; Siracusa et al. 2020). In an HHcy model, we previously showed the protective benefits of cashews (D'Amico et al. 2022). Specifically, since HHcy results in the malfunction of several organs, like the kidney, liver, or intestine, we found that daily cashew ingestion might reverse tissue abnormalities, oxidative stress, and the release of proinflammatory cytokines. This study extends our previous work by investigating brain‐specific neuroinflammation/neuroprotection. The data come from an independent cohort using the same HHcy protocol and cashew dosage; but no animals or samples were reused. This study explicitly focuses on the brain and demonstrates CNS‐specific mechanisms—activation of Nrf2/HO‐1, preservation of MAP‐2, reduction of Iba1, GFAP, and neuronal death—that had not been previously identified, justifying a separate study.

Therefore, the purpose of this study was to examine the protective effects of cashews against HHcy‐induced brain changes in rats by looking at biomarkers for Hcy, oxidative stress, and neuroinflammation.

## 2 Materials and Methods

### 2.1 Cashew Nuts Nutritional Composition

The cashew kernel samples (*Anacardium occidentale L*.) were obtained from the Ivory Coast. The nutritional composition of cashew was evaluated according to the Association of Official Analytical Chemists (AOAC) Official Method as reported previously (Cordaro, Fusco, et al. 2020; D'Amico et al. 2022) (see table 1; Siracusa et al. 2020). The total content of folate in cashew nuts is 25 μg/100 mg (https://fdc.nal.usda.gov/fdc‐app.html#/food‐details/170162/nutrients, accessed on 5 March 2022). Cashew kernel samples were ground and then dissolved in saline prior to oral administration, freshly prepared each day (Siracusa et al. 2020). Rats were administered cashew nuts at a dose of 100 mg/kg by oral gavage.

### 2.2 HHcy Induction and Experimental Groups

Sprague Dawley rats (male, 250 g, Envigo, Milan, Italy) were housed in a well‐organized environment and fed normal rodent food and water. The Animal Welfare Review Board at Messina University gave its approval to the study (ethical protocol code: no. 897/2021‐PR). All animal research complies with EU regulations (EU Directive 2010/63) and new Italian legislation (D.Lgs 2014/26).

HHcy was induced by administration of methionine (Meth) at the dose of 1 g/kg. Meth powder was weighed and dissolved in drinking water, freshly prepared each day for 30 days (D'Amico et al. 2022; Ansari and Bhandari 2008).

The animals were randomly distributed into groups:

− Sham controls: rats received only normal saline;
− HHcy+vehicle: rats received Meth as above and were treated with saline for 30 days;
− HHcy+cashew nuts: rats received Meth as above, and were treated with oral cashew nuts (100 mg/kg) for 30 days.

The number of animals was calculated using G*power; 15 animals/group were used, divided into five for each technique. The dose of cashew nuts was chosen based on a preliminary study which showed that the dose of 100 mg/kg was the most effective dose (Siracusa et al. 2020).

Since no significant difference was found between sham groups, only data regarding sham+vehicle groups were shown. Rats were sacrificed after 30 days by cervical dislocation. Blood and brain tissues were taken from all animals.

### 2.3 Biochemical Analyses

Levels of Hcy in serum were assessed using a commercial kit for HPLC measurements (Bio‐Rad, Milan, Italy) (D'Amico et al. 2022).

### 2.4 Glutathione (GSH) Levels

GSH levels were determined in blood according to manufacturer's instructions (Cusabio Biotech Co. Ltd., China) (Cordaro, Siracusa, et al. 2020). The levels of GSH in brain tissues were measured as previously described method (Samarghandian et al. 2016).

### 2.5 Malondialdehyde (MDA) Levels

Plasma MDA and brain MDA levels were determined as previously described (D'Amico et al. 2022; Cuzzocrea et al. 2000; Cordaro, Salinaro, et al. 2021).

### 2.6 Western Blots for NRF‐2, HO‐1, Bax and Bcl‐2

Cytosolic and nuclear extracts were prepared as previously described (D'Amico et al. 2021; Cordaro, Trovato, et al. 2021). Protein concentration was estimated by the Bio‐Rad protein assay (Bio‐Rad, 5000006, Biogenerica SRL, CT, Italy) using bovine serum albumin (a9647, Sigma Aldrich, St. Louis, MO, USA) as standard. The following primary antibodies were used: anti‐NRF‐2 (sc‐365949, Santa Cruz Biotechnology (SCB); 1:500), anti‐HO‐1 (sc‐136,960, SCB; 1:500), anti‐Bcl‐2 (sc‐7382, SCB; 1:500), anti‐Bax (sc‐7480, SCB; 1:500), in phosphate‐buffered saline at 4°C overnight. As controls, Anti‐β‐actin or anti‐lamin A/C antibodies were used. The expression of protein bands was detected by a procedure previously described (Siracusa et al. 2021).

### 2.7 Immunohistochemical Analysis

Immunohistochemical analysis was performed as previously described (Cordaro, Salinaro, et al. 2021; Peritore et al. 2021). The sections were incubated overnight with primary antibodies: anti‐GFAP (sc‐51908, SCB), anti‐Iba‐1 (sc‐32725, SCB), anti‐microtubule‐associated protein 2 (MAP‐2; sc‐74421, SCB). Images were collected using a Leica DM6 microscope (Leica Microsystems SpA, Milan, Italy) following a typical procedure. The histogram profile is related to the positive pixel intensity value obtained.

### 2.8 Terminal Deoxynucleotidyl Nick‐End Labeling (TUNEL) Assay

Apoptosis was analyzed by a TUNEL assay using cell death detection kit. TUNEL labeling for apoptotic cell nuclei was carried out according to the previously mentioned protocol (Genovese et al. 2021).

### 2.9 Statistical Evaluation

All values are expressed as mean ± standard error of the mean (SEM) of N observations. The pictures displayed are typical of at least three tests conducted on several experimental days using tissue sections taken from every animal in each group. For in vivo studies, *N* represents the number of animals used. The results were analyzed by one‐way ANOVA followed by a Bonferroni post hoc test for multiple comparisons. *P* value < 0.05 was considered significant.

### 2.10 Materials

Methionine (purity ≥ 99%) was purchased from Sigma Chemical (St. Louis, MO, USA). All chemicals were taken from Sigma‐Aldrich, and stock solutions were prepared in saline (0.9% NaCl; Baxter, Milan, Italy).

## 3 Results

### 3.1 Valuation of Serum Hcy, Plasma MDA and Plasma GSH Levels After HHcy

**FIGURE 1:**
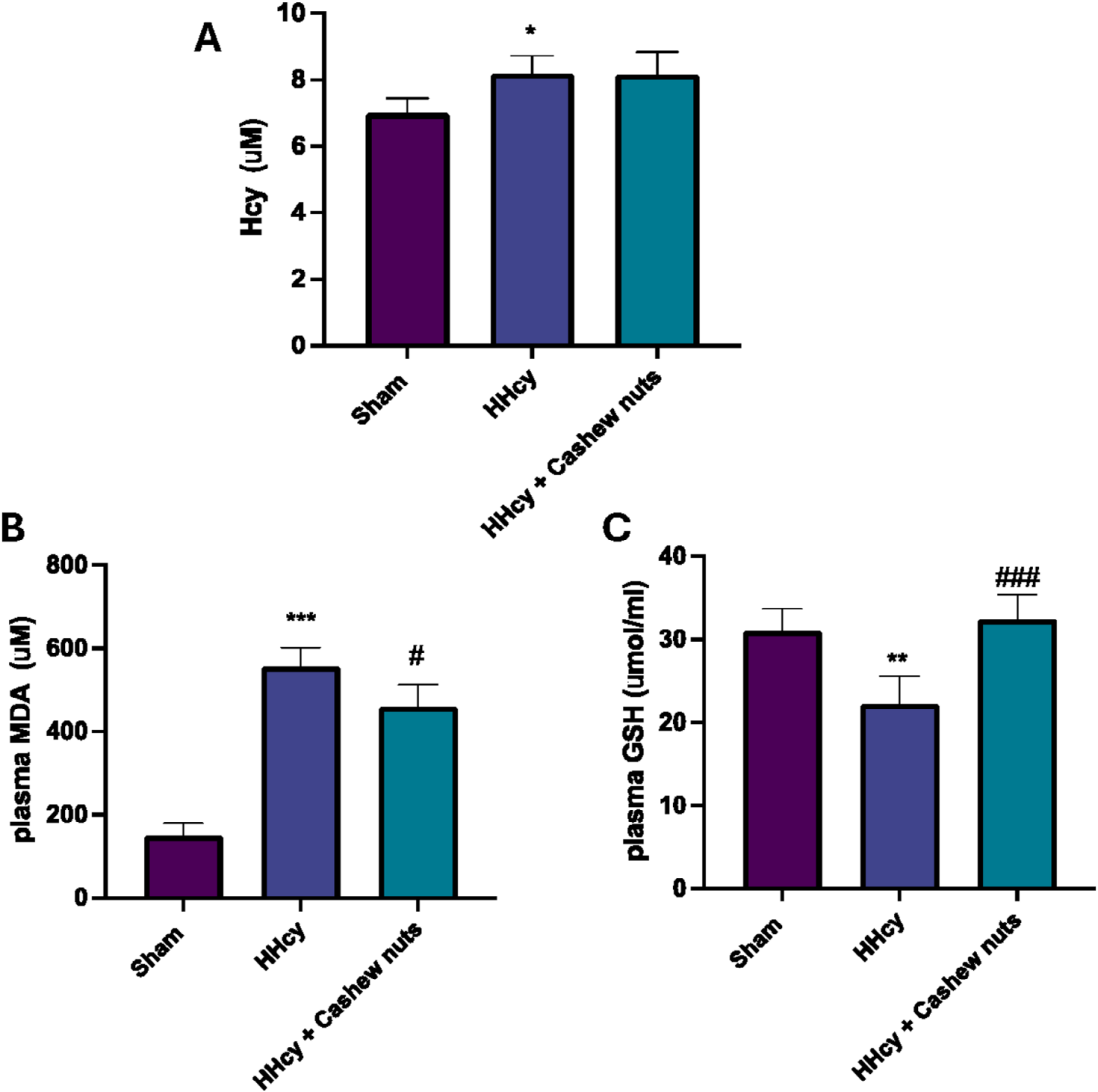
Evaluation of Hcy, MDA, and GSH levels. Serum levels of Hcy (A). Plasma levels of MDA (B) and GSH (C). Values are means ± SEM of five animals for each group. *p < 0.05 versus sham; **p < 0.01 versus sham; ***p < 0.001 versus sham; ^#^ *p* < 0.05 versus HHcy; ^###^ *p* < 0.001 versus HHcy.

First of all, to see whether daily administration of Meth effectively caused a status of HHcy, we measured in the serum the levels of Hcy. Oral Meth administration at the dose of 1 g/kg oral for 30 days caused an increased level of Hcy compared to the control (Table 1) group (Figure 1A). Treatment with Cashew nuts (100 mg/kg) was unable to directly reduce elevated serum Hcy levels (Figure 1A); this suggests that the possible protective effect of cashews could be due to the modulation of oxidative stress induced by an HHcy condition.

**TABLE 1:** Nutritional composition of cashew nuts.

Component	Value per 100 g
Moisture	5.40 g
Protein	22.46 g
Total lipids	44.19 g
Dietary fiber	4.48 g
Total sugars	30.95 g
Ash	2.68 g
Total phenols	80.01 mg

To confirm this, we observed elevated plasma levels of MDA (Figure 1B), a marker of lipid peroxidation, and a significant reduction in plasma GSH levels in HHcy rats compared to sham animals (Figure 1C). On the contrary, cashew nuts significantly reduced MDA levels and upregulated GSH levels in plasma (Figure 1B,C, respectively), thus modulating oxidative stress.

### 3.2 Valuation of Oxidative Stress in Brain Induced by HHcy

**FIGURE 2:**
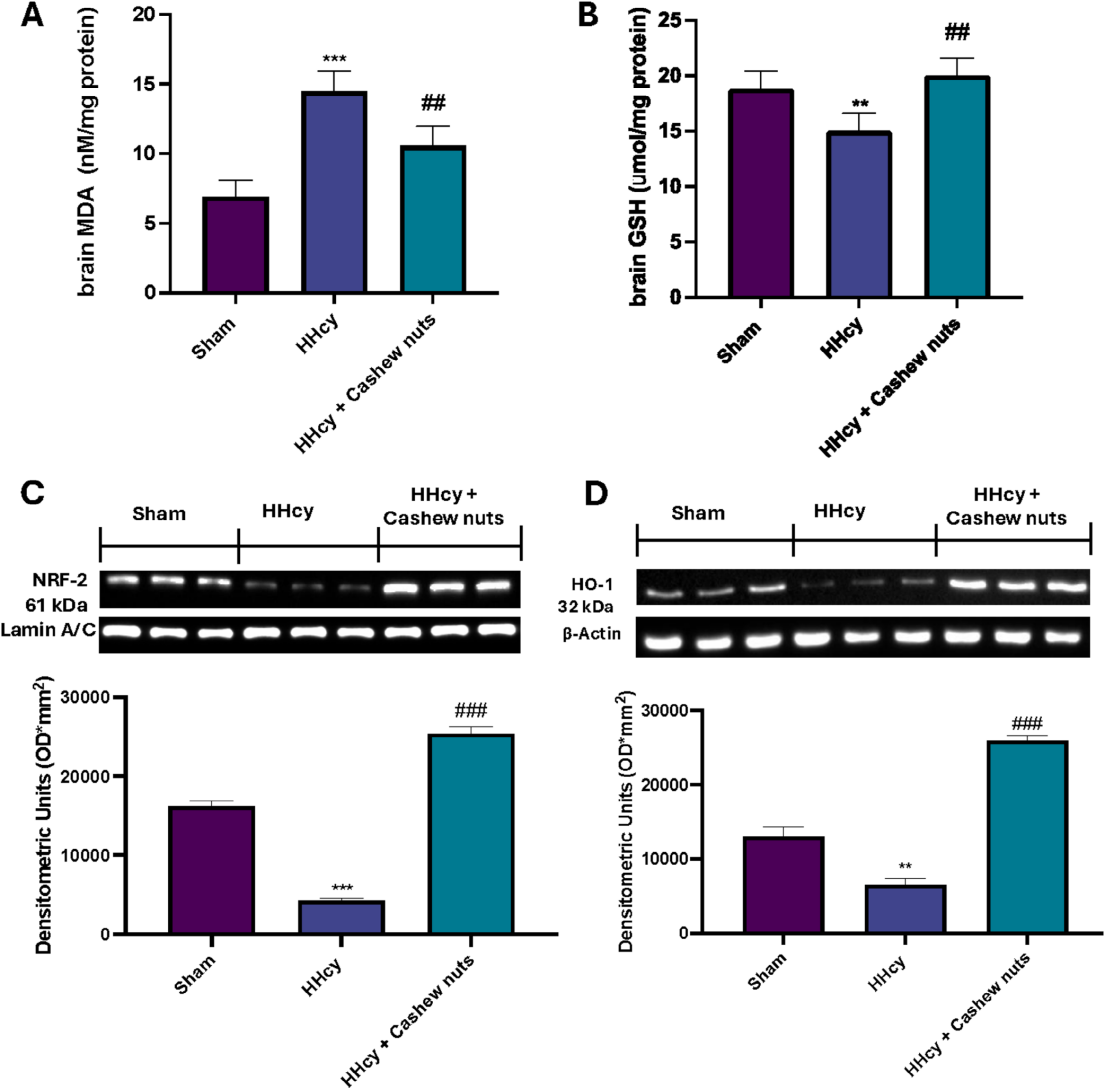
Evaluation of oxidative stress in brain. MDA (A) and GSH (B) levels in brain tissue. Western blot and relative densitometric analysis: Nrf2 (C) and HO‐1 (D). Exposed is a blot of lysates (five animals/group) with a densitometric analysis for all animals. Values are means ± SEM of five animals for each group. **p < 0.01 versus sham; ***p < 0.001 versus sham; ^##^ *p* < 0.01 versus HHcy; ^###^ *p* < 0.001 versus HHcy.

Furthermore, since the brain is one of the organs that are altered following HHcy, we investigated the involvement of oxidative stress also in the brain. Our results showed an increase in MDA levels(Figure 2A) and a significant reduction in GSH levels (Figure 2B) in brain samples taken from the HHcy group compared to control. Similarly, we observed a modulation of the Nrf2/HO‐1 pathway, as demonstrated by western blot analysis, by reduced expression of Nrf2 (Figure 2C) and HO‐1 (Figure 2D) in HHcy animals. Daily consumption of cashew nuts reduced MDA levels (Figure 2A) in the brain as well as upregulated GSH levels and Nrf2 and HO‐1 expressions (Figure 2B,C,D, respectively), confirming its antioxidant properties.

### 3.3 Valuation of Markers Related to Neuroinflammation Induced by HHcy

**FIGURE 3:**
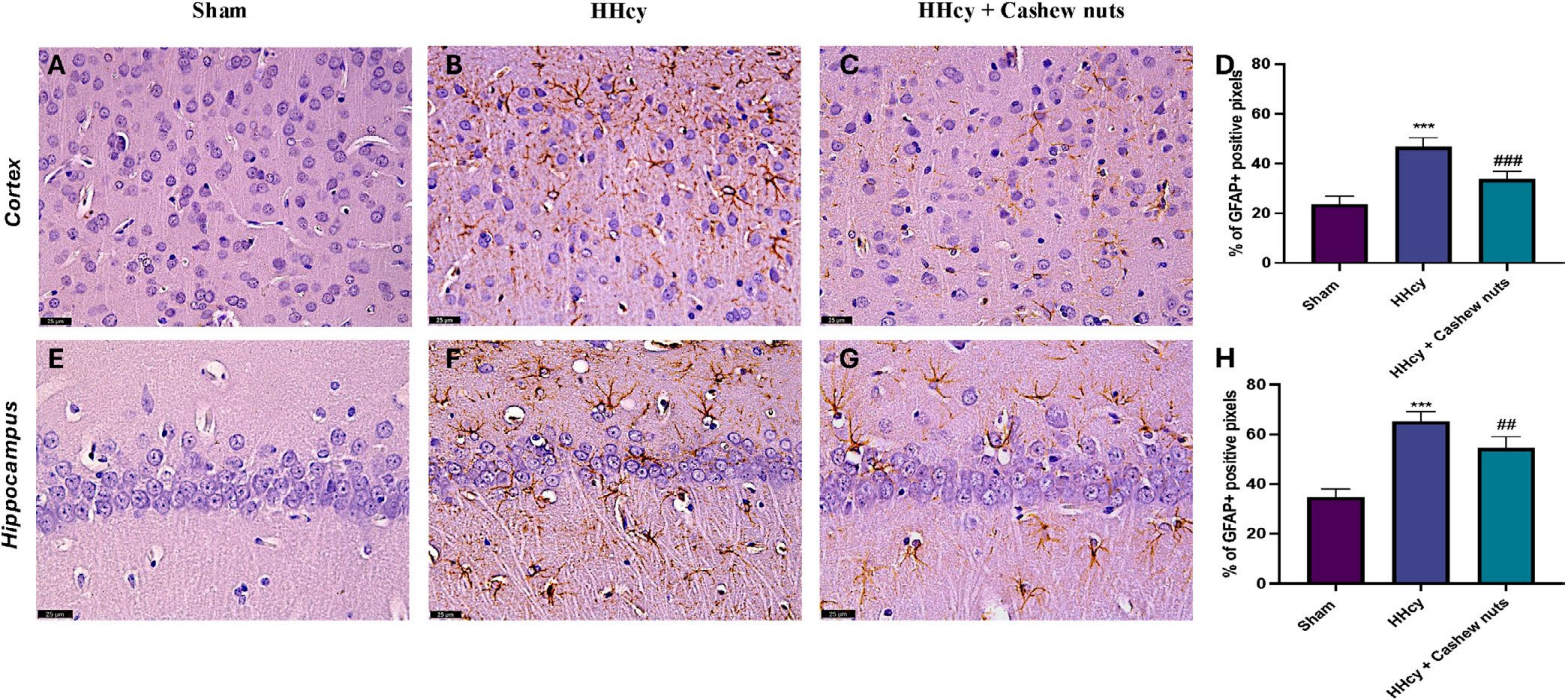
Evaluation of GFAP expression in brain section. Immunohistochemical analysis for GFAP in cortex area: Sham (A), HHcy (B), HHcy + Cashew nuts (C), graphical quantification (D). Immunohistochemical analysis for GFAP in CA1 hippocampus area: Sham (E), HHcy (F), HHcy + Cashew nuts (G), graphical quantification (H). A 40× magnification is shown (25‐μm scale bar). Values are means ± SEM of five animals for each group. ***p < 0.001 versus sham; ^##^ *p* < 0.01 versus HHcy; ^###^ *p* < 0.001 versus HHcy.

**FIGURE 4:**
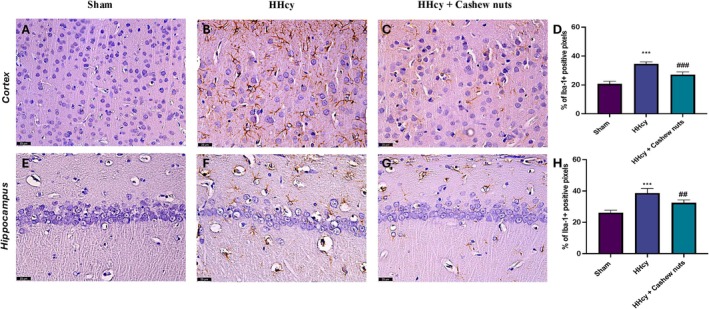
Evaluation of Iba‐1 expression in brain section. Immunohistochemical analysis for Iba‐1 in cortex area: Sham (A), HHcy (B), HHcy + Cashew nuts (C), graphical quantification (D). Immunohistochemical analysis for Iba‐1 in CA1 hippocampus area: Sham (E), HHcy (F), HHcy + Cashew nuts (G), graphical quantification (H). A 40× magnification is shown (25 μm scale bar). Values are means ± SEM of five animals for each group. ***p < 0.001 versus sham; ^##^ *p* < 0.01 versus HHcy; ^###^ *p* < 0.001 versus HHcy.

To assess the neuroinflammation, we evaluated the expression of GFAP and Iba‐1, markers of astrocytes and microglia activation, by immunohistochemical analyses. Our results showed an important increase in GFAP expression both in the cortex and CA1 area of the hippocampus in HHcy group (Figure 3,B,D,F,H, respectively), compared to sham animals (Figure 3A,D,E,H respectively). At the same way, we observed a significant positive staining for Iba‐1 both in the cortex and CA1 area of the hippocampus in HHcy rats (Figure 4B,D,F,H, respectively), compared to the control group (Figure 4A,D,E,H respectively).

Orally administrations of Cashew nuts significantly reduced both markers of neuroinflammation (Figure 3C,D,G,H; for GFAP and Figure 4C,D,G,H for Iba‐1).

### 3.4 Valuation of MAP2 Expression, a Neuronal Marker, After HHcy

**FIGURE 5:**
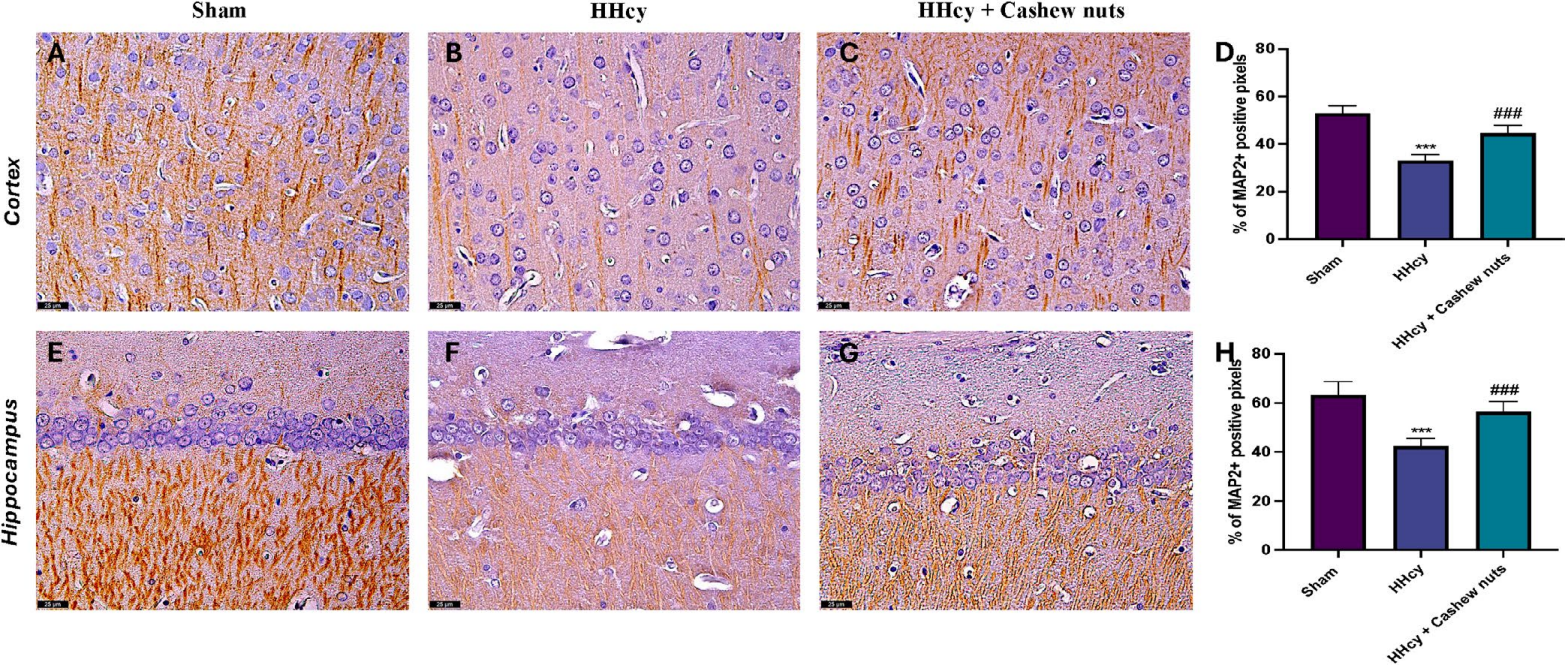
Evaluation of MAP2 expression in brain section. Immunohistochemical analysis for MAP2 in cortex area: Sham (A), HHcy (B), HHcy + Cashew nuts (C), graphical quantification (D). Immunohistochemical analysis for MAP2 in CA1 hippocampus area: Sham (E), HHcy (F), HHcy + Cashew nuts (G), graphical quantification (H). A 40× magnification is shown (25 μm scale bar). Values are means ± SEM of five animals for each group. ***p < 0.001 versus sham; ^###^ *p* < 0.001 versus HHcy.

MAP 2 is the predominant cytoskeletal regulator within neuronal dendrites, necessary to maintain neuroarchitecture (DeGiosio et al. 2022). Consistent with the literature, sections taken from the sham group displayed an important positive MAP2 immunostaining both in the cortex and CA1 area of the hippocampus (Figure 5A,D,E,H, respectively); on the contrary, MAP2 expression was significantly reduced after Meth administration both in the cortex and CA1 area of the hippocampus (Figure 5B,D,F,H, respectively). Cashew nuts were able to partially restore MAP2 expression (Figure 5C,D,G,H, respectively).

### 3.5 Effect of Cashew Nuts on Neuronal Death Induced by HHcy

**FIGURE 6:**
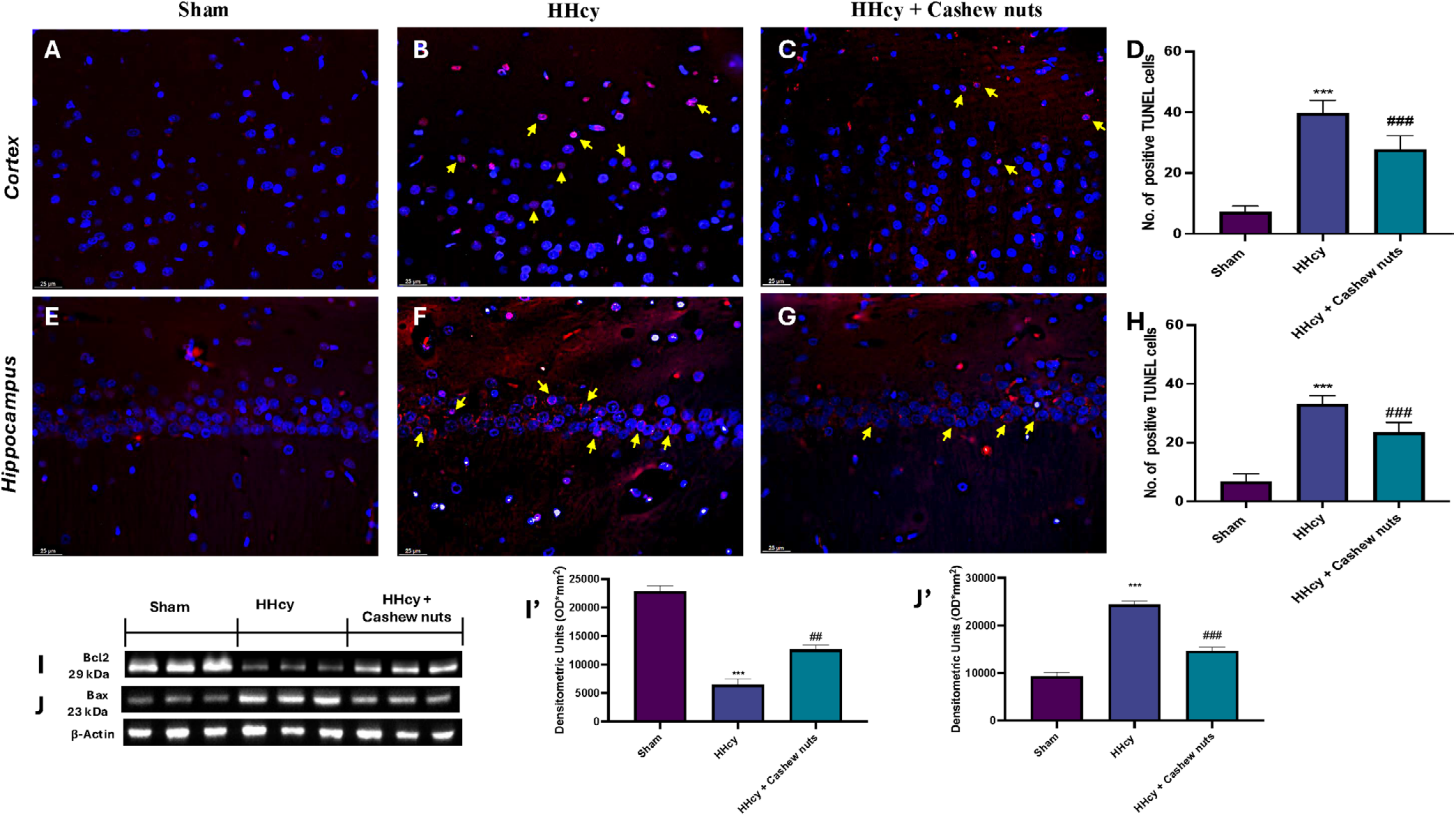
Evaluation of apoptotic process. TUNEL assay in cortex area: Sham (A), HHcy (B), HHcy + cashew nuts (C), number of positive cells per high‐power field (D). TUNEL assay in CA1 hippocampus area: Sham (E), HHcy (F), HHcy + cashew nuts (G), number of positive cells per high‐power field (H). A 40X magnification is shown (25 μm scale bar). Western blot and relative densitometric analysis: Bcl‐2 (I, I') and Bax (J, J'). Exposed is a blot of lysates (five animals/group) with a densitometric analysis for all animals. Values are means ± SEM of five animals for each group. ***p < 0.001 versus sham; ^##^ *p* < 0.01 versus HHcy; ^###^ *p* < 0.001 versus HHcy.

To evaluate whether the apoptosis pathway is involved in damage of HHcy induced by Meth, we performed the TUNEL assay on brain sections. In comparison to the sham group (Figure 6A,D,E,H, respectively), our results demonstrated a significantly higher number of TUNEL‐positive cells in the cortex and CA1 area of the hippocampus in HHcy rats (Figure 6B,D,F,H, respectively). Administration of cashew nuts resulted in a significant reduction in the quantity of apoptotic cells (Figure 6C,D,G,H, respectively). We verified this by looking at brain samples' expression of the anti‐apoptotic Bcl‐2 and pro‐apoptotic Bax proteins. In the HHcy group, Bcl‐2 levels were considerably lower (Figure 6I,I'); nevertheless, following meth use, Bax expression was increased (Figure 6J,J'). Both markers returned to levels comparable to those observed in the sham group after cashew nut treatments.

## 4 Discussion

HHcy occurs when blood Hcy levels are above normal limits, due to a disruption in Hcy metabolism (Veeranki et al. 2017). The main causes of HHcy are either nutritional shortages of vitamins B6, B12, choline, and folate, which are cofactors for the enzymes, or genetic defects in the metabolism‐related enzymes that remethylate or transsulfurate Hcy (Fu et al. 2018). Hcy has prooxidant activity; hence, HHcy‐induced oxidative stress may arise as a result of reduced activity of antioxidant enzymes as well as increased formation of ROS (Jacobsen 2000). In fact, it has been documented that HHcy stimulates the generation of hydroxyl radicals, which start the process of lipid peroxidation. MDA and 4‐hydroxy‐2‐nonenal are byproducts of this process, which can cause lipids, proteins, and nucleic acids to oxidize (Çelik et al. 2017). Elevated Hcy levels can suppress the production of enzymes like GSH peroxidase (GPx), a potent antioxidant that neutralizes ROS (Lubos et al. 2007; Ismail et al. 2022). In this regard, several recent evidence suggests that mild HHcy, whether baseline or transient following a methionine load, compromises endothelial cell functions by producing ROS and inflammation, which in turn raises the risk of stroke and cerebrovascular issues (Baloghova et al. 2022; Wang et al. 2021; Kaplan et al. 2020; Yang et al. 2010).

The oxidative effect could be neutralized through the consumption of products rich in polyphenols, such as vegetables and dried fruit. One of the four most well‐known nuts in the world is the cashew, whose tree, *Anacardium Occidentale L*., is used in traditional medicine as a high‐nutrient, edible nut with a high content of mineral components, anthocyanins, carotenoids, flavonoids, folates and other polyphenols (Cordaro, Siracusa, et al. 2020). In this work, we showed that daily cashew nut consumption could reduce neuroinflammation and oxidative stress in Meth‐induced HHcy rats. Our results are consistent with previous research that shown that meth supplementation decreased antioxidant enzyme activity, increased plasma Hcy levels, and induced oxidative stress (D'Amico et al. 2022; Luo et al. 2014; Alfeel et al. 2023; Alghadir et al. 2021). In contrast, oral administration of cashews (100 mg/kg) reduced lipid peroxidation and upregulated GSH levels in blood system, confirming the antioxidant proprieties of cashew nuts (Figure 1). This model of HHcy is a commonly used experimental model for detecting the neurotoxic impact of elevated levels of Hcy and is intended to partially replicate conditions found in the general adult population where the mild HHcy prevalence is approximately 5%–7% (Kovalska et al. 2023). Meth‐rich diets have been demonstrated to change brain activity and start neurotoxic effects, which lead to increased neuroinflammation and noticeable cognitive deficits (Nuru et al. 2018; Weekman et al. 2019). Additionally, clinical research shows that HHcy causes autophagy, apoptosis, synaptic remodeling, memory loss, dementia, and cerebrovascular micro‐hemorrhages (Kovalska et al. 2021; Cao et al. 2021); for this reason, HHcy is correlated with Alzheimer's disease with the presence of amyloid plaques accumulated around the microvessels, as well as slightly elevated hyperphosphorylated tau (Alachkar et al. 2022; Tapia‐Rojas et al. 2015). However, the precise toxic process affecting neuronal tissue is still incompletely understood. Several studies demonstrated that high blood Hcy levels led in rats to the alteration of brain tissue in the cerebral cortex as well as the hippocampus (Kovalska et al. 2020, 2019, 2018). Particularly vulnerable to neurodegenerative processes that impact the various kinds of plasticity, learning, and memory is the hippocampus (Kovalska et al. 2023). In fact, compared to CA3 and dentate gyrus, hippocampus neurons in the CA1 region are extremely vulnerable to insults like ischemia, inflammation, hypoglycemia, or excitotoxicity. This makes it one of the most vulnerable brain regions, and it has been investigated for declines in the mechanisms of development and progression of neurodegeneration (Kovalska et al. 2021; Ugolini et al. 2018; Radenovic et al. 2020). Other studies' findings showed that mild HHcy caused histomorphological changes in the brain and hippocampus, including changes in the quantity and shape of astrocytes and microglia (Kovalska et al. 2020). Our work is in line with previous research, as observed by an increased activation of astrocytes and microglia by immunohistochemistry technique with a significant increase in positive staining for GFAP and Iba‐1 in the Meth‐treated group, while daily treatment with cashews was able to modulate astrocyte and microglial activation (Figures 3 and 4, respectively). At the same time, MAP2 expression was significantly reduced after Meth intake for 4 weeks. MAP‐2 is a static, structural protein, that is essential for maintaining neuroarchitecture, along with other cytoskeletal proteins. Due to its exceptional sensitivity to a wide range of stimuli, MAP‐2 has been shown to play dynamic roles in the development, differentiation, and plasticity of neurons. These functions include important involvement in the responses of neurons to growth factors, neurotransmitters, synaptic activity, and neurotoxins (DeGiosio et al. 2022; Soltani et al. 2005; Zhao et al. 2019). Our findings are consistent with previous research, as evidenced by immunohistochemical analysis and the restoration of MAP2 expression following cashew nut treatment (Figure 5). This neuroprotective effect of cashews probably resulted from a significant modulation of oxidative stress, not only at a systemic level but also at a brain level, as demonstrated by a reduction in MDA levels and significant upregulation of Nrf2, HO‐1 and GSH in brain collected from cashew‐treated rats (Figure 2). Additionally, experiments with on Meth intake on mice or rats demonstrated an increased number of TUNEL+ cells with the increase in apoptosis rates, DNA damage and caspase activity (Kovalska et al. 2021; Soares et al. 2017). Consistently, our results detected increased neuronal cell death stained with TUNEL assay specifically in the CA 1 region of the hippocampus and cortex, in the Meth group compared to control animals. Moreover, HHcy also induced a several alterations of apoptotic markers, such as Bax and Bcl‐2 (Figure 6). Here we demonstrated that treatment with cashews is able to reduce the apoptotic process, suggesting a protective action due to cashew intake.

Overall, these neuroprotective results are likely due to the nutritional composition of cashew nuts, particularly folate vitamers and polyphenols. Indeed, folates provide a direct biochemical link to Hcy management. By facilitating the remethylation of Hcy to methionine, dietary folates counteract the state of redox disruption induced by meth loading. This is consistent with the brain model we documented, as demonstrated by the upregulation of Nrf2/HO‐1 and the restoration of redox‐sensitive indices, indicating a shift toward increased antioxidant capacity. Second, the polyphenolic fraction offers a plausible pathway for controlling neuroinflammation and limiting apoptosis. A broad body of literature supports polyphenol‐mediated Nrf2 activation (Ebrahimi et al. 2025; Jin et al. 2023; Kanner 2023). This is consistent with our data, in which polyphenols attenuate glial activation and pro‐apoptotic signaling, with both pathways likely converging toward Nrf2‐centered cytoprotection.

### 4.1 Limitations of Study

This study has some limitations. First, meth‐induced HHcy is a practical model, but it does not fully replicate human HHcy. Second, the model is based exclusively on male subjects, a choice justified by the fact that sex hormones modulate HHcy metabolism and microglial reactivity; this is consistent with previous literature on HHcy (Rossokha et al. 2023; Niu et al. 2022; Randeva 2003; Davis et al. 2026; Ocanas et al. 2023). For dietary translation, a simple allometric conversion (human equivalent dose≈rat dose × 0.162) indicates that the tested intake corresponds to a modest daily amount in adults (Nair and Jacob 2016; Nair et al. 2018); however, food matrices and feeding patterns differ between species, so this estimate should be interpreted with caution and validated in human clinical trials. Indeed, excessive cashew consumption may be associated with several problems. Real‐world use carries the risk of allergies and possible exposure to oxalate, which should be considered in dietary guidelines. Furthermore, the lack of data on lipids and liver enzymes is a limitation, given the nut's energy density and its rich profile in monounsaturated fatty acids (MUFAs). Future studies incorporating behavioral/cognitive data, female cohorts, dose–response analyses, and metabolic safety panels will be essential to define both efficacy boundaries and translational safety.

## 5 Conclusions

Given its significance in the etiology of neurological diseases and its rising prevalence in the global population, HHcy is a stimulating topic for further study. Our research demonstrates that elevated Hcy causes ROS generation, morphological changes in the cortex and hippocampus, and neuronal death, all of which contribute to cellular dysfunction and tissue damage. The present results indicate that cashews significantly reduced neuroinflammation and apoptosis in hyperhomocysteinemic rats. Cashews' neuroprotective effects are likely due to their antioxidant qualities, which lower lipid peroxidation and boost the activity of antioxidant enzymes like GSH in plasma. Thus, the balanced consumption of cashew nuts could have beneficial action in the prevention of HHcy‐induced disorders. Although the improvement from a brain perspective is a promising result, further studies are needed to broaden knowledge not only at the basic research level, but also in the clinical setting.

## Author Contributions

Conceptualization, D.I. and R.D.; methodology, A.A. and R.A.; validation, S.C., R.D.P.; formal analysis, M.C. and R.F.; data curation, R.S. and G.M.; writingoriginal draft preparation, R.S. and A.A.; writingreview and editing, D.I., R.D., S.C.; supervision, D.I. and R.D.P. All authors have read and agreed to the published version of the manuscript.

## Funding

The authors have nothing to report.

## Ethics Statement

This study was approved by the University of Messina Review Board for the care of animals (ethical protocol code: no. 897/2021‐PR).

## Consent

The authors have nothing to report.

## Conflicts of Interest

The authors declare no conflicts of interest.

## Data Availability

All data included in this study are available upon request by contact with the corresponding author.
